# Supplementary material for: Growth of soil ammonia-oxidizing archaea on air-exposed solid surface
Source: ISME Commun. 2024 Oct 24;4(1):ycae129. doi: 10.1093/ismeco/ycae129 (PMC11561398; doi:10.1093/ismeco/ycae129)
Supplement: Supplementary_information_ycae129 [file supplementary_information_ycae129.docx]

# **Supplementary Information for**

**Growth of soil ammonia-oxidizing archaea on air-exposed solid surface**

Running title: Growth of soil AOA on solid surface

Christiana Abiola^a^, Joo-Han Gwak^a^, Ui-Ju Lee^a^, Samuel Imisi Awala^a^, Man-Young Jung^bcd^, Woojun Park^e^, and Sung-Keun Rhee^a,^*

^a^Department of Biological Sciences and Biotechnology, Chungbuk National University, 1 Chungdae-ro, Seowon-Gu, Cheongju 28644, Republic of Korea.

^b^Interdisciplinary Graduate Programme in Advance Convergence Technology and Science, Jeju National University, Jeju, Republic of Korea.

^c^Department of Science Education, Jeju National University, Jeju, Republic of Korea.

^d^Jeju Microbiome Center, Jeju National University, Jeju, Republic of Korea.

^e^Laboratory of Molecular Environmental Microbiology, Department of Environmental Science and Ecological Engineering, Korea University, Anam-Dong, Seungbuk-Ku, Seoul 02841, Republic of Korea.

***Corresponding author**: Sung-Keun Rhee

Mailing address: Department of Biological Sciences and Biotechnology, Chungbuk National University, 1 Chungdae-ro, Seowon-Gu, Cheongju 28644, Republic of Korea.

Email: [rhees@chungbuk.ac.kr](mailto:rhees@chungbuk.ac.kr) Phone: +82-43-261-2300. Fax: 82-43-264-9600.

**The file includes:**

Supplementary Notes 1 and 2

Supplementary Figures S1 to S8

Supplementary Tables S1, S2 and S10

Legends for Supplementary Tables S3 to S9, S11 to S14

Supplementary References.

**Supplementary Note 1**

## **Transcriptome analysis of N. viennensis EN76.**

***Ammonium transport and Cell division:*** Two of the ammonium transporters (*amt1*; NVIE_RS01185, and *amt3*; NVIE_RS11415) were downregulated in *N.* *viennensis* EN76 floating filter-grown cells, while *amt2* (NVIE_RS10630) showed constitutive expression (**Supplementary Table S6**). These results correspond with the lower growth rates and biomass production of *N.* *viennensis* EN76 cells grown on floating filters compared to the control culture in liquid media. The Cdv system is the primary cell division system in archaea, as evidenced in a marine ammonia-oxidizing archaeon, *Nitrosopumilus* *maritimus* SCM1 [1]. We observed that the putative archaeal cell division genes (*cdvB*; NVIE_RS02340 and *cdvC*; NVIE_025520) were downregulated in *N.* *viennensis* EN76 floating filter-grown cells (**Supplementary Table S8**). In contrast, other genes of the Cdv system such as *cdvA* (NVIE_RS00655), other *cdvB* homologs (*snf7*; NVIE_RS09745, *snf7*; NVIE_RS04040, *snf7*; NVIE_RS09110, and *snf7*; NVIE RS12195), and *ftsZ* (NVIE_RS05305) were constitutively expressed (**Supplementary Table S8**). Caspi et al. [2] reported that *cdvB* and *cdvC* are crucial for the endosomal sorting complex required for transport (ESCRT) pathway, which is involved in various cell division processes. Therefore, downregulation of the *cdvC*, which encodes an ATPase essential for the turnover of ESCRT membrane-abscission polymers [2], may lead to reduced turnover of these polymers, consequently affecting the regulation of controlled division of cells grown on floating filters.

**Supplementary Note 2**

# **Transcriptomic analysis of *N. europaea* ATCC 19718**

**T**he key genes involved in ammonia oxidation activity and energy conservation showed constitutive expression in *N. europaea* ATCC 19718 (**Supplementary Table S11**). Among the 70 genes that exhibited significant differential expression **(**log_2_FC > 1 and FDR < 0.05), 53 were upregulated, and 17 were downregulated in *N. europaea* ATCC 19718 floating filter-grown cells (**Supplementary Table S12**).

Among the sigma-70 (σ 70) families, group IV, also known as the extracytoplasmic function sigma factor (σ ECF) subfamily, is the largest and most diverse subfamily [3, 5]. A phylogenetic analysis of the nine upregulated σ 70 sequences in cells of *N. europaea* ATCC 19718 grown on floating filters (**Fig. S8**) revealed their clustering within the σ ECF subfamily. Cell-surface signaling, a signaling cascade that starts at the outer membrane and ends in the cytoplasm, was first described for the FecI/FecR pair in iron acquisition in *E. coli* [6]. The regulatory system was expanded to cell envelop-related processes such as biofilm formation and iron metabolism [6–9] This finding aligns with our observation in floating filter-grown cells of *N. viennensis* EN76, where the genes for EPS biosynthesis and iron homeostasis were upregulated. Together, the extracytoplasmic stress response mediated by the σ ECF subfamily in *N. europaea* ATCC 19718 cells grown on floating filters might be successfully used to sustain ammonia oxidation activity and growth of *N*. *europaea* ATCC 19718, as observed in **Fig. 1E**.


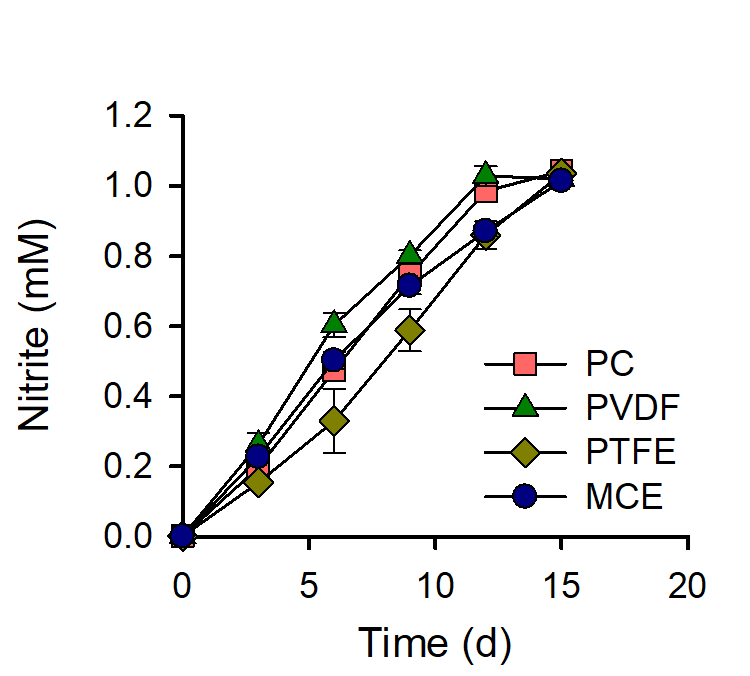


**Figure S1.** Ammonia oxidation activity of *N.* *viennensis* EN76 cells grown on different filters. The filters used include PC: polycarbonate, PVDF: polyvinylidene fluoride, PTFE: polytetrafluoroethylene, and MCE: mixed cellulose esters. An inoculum size of 10^7^ cells was used for the experiment. All experiments were performed in triplicates. Data are presented as mean ± SD *(n*=3), and the error bars are hidden when they are smaller than the width of the symbols.

**Figure S2.** Ammonia oxidation activity of *N.* *viennensis* EN76 cells grown on floating filters and liquid media. The liquid media was either in cell culture flasks or petri dishes. An inoculum size of 10^7^ cells was used for the experiment. All experiments were performed in triplicates. Data are presented as mean ± SD (*n*=3), and the error bars are hidden when they are smaller than the width of the symbols.


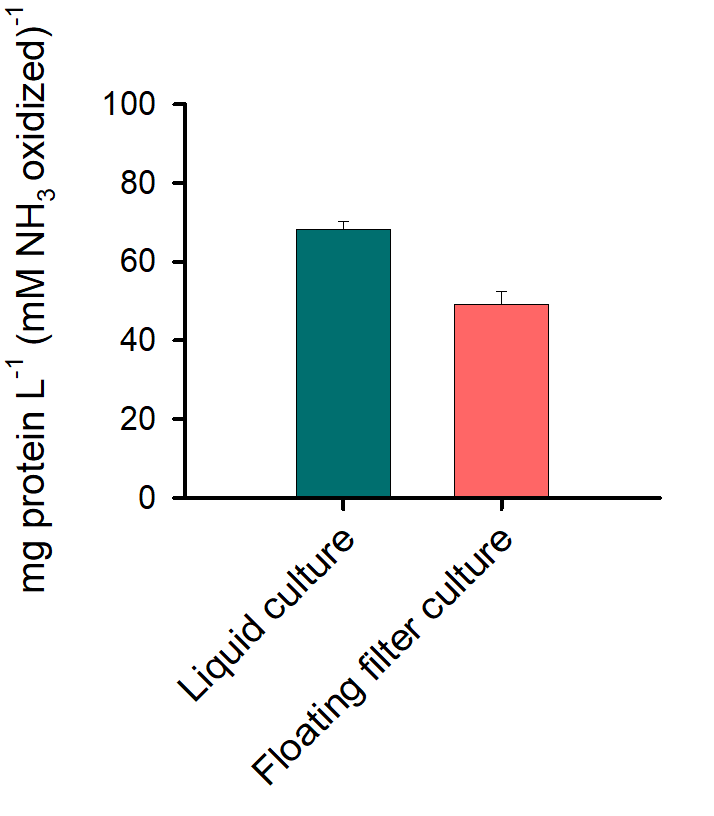


**Figure S3.** Growth yield of *N. viennensis* EN76 cells grown on floating filters and liquid media. The yield was calculated as total cellular proteins produced after complete oxidation of 1 mM ammonia. All experiments were performed in triplicates. Data are presented as mean ± SD (*n*=3).

**Figure S4.** Ammonia oxidation activity of ‟*N. chungbukensis*” MY2 cells inoculated on floating filters using vacuum and ambient gravitational force filtration. Ammonia oxidation in the control culture grown in liquid media was used for comparison. All experiments were performed in triplicates. Data are presented as mean ± SD (*n*=3), and the error bars are hidden when they are smaller than the width of the symbols.

**Figure S5.** Comparative gene expression pattern of *N.* *viennensis* EN76 cells grown on floating filters and liquid media. Each bar in the functional class shows the number of upregulated genes. Genes are assumed to be differentially expressed when the log_2_FC > 1 and FDR < 0.05. Transcriptome experiments were performed in triplicates.

**Figure S6.** Alignment of amino acid sequence of multicopper oxidases (MCOs) showing the copper-binding sites in both two-domain (2dMCOs) and three-domain (3dMCOs) MCOs. Sequences of 2dMCOs were clustered into three types: A, B, and C. Two sequence fragments from the N-terminal and C-terminal of the first and the last domains, respectively, are indicated. The numbers above and alongside each sequence fragment correspond to the residue positions. The turquoise and green colours in the alignments indicate the consensus positions of the copper-binding residues and the trinuclear histidine binding sites, respectively. Residues with the red-colored triangles below them are of T1 copper sites. Residues with purple-colored triangles below them are trinuclear binding histidine. The sequence ID (NCBI accession number) and the origin of each sequence are listed. This figure was modified from Figure 1 in [10].

**Figure S7.** Alignment of the amino acid sequences of the Cop proteins (CopC*/*CopD NVIE_RS06945 and NVIE_RS06955) from *N. viennensis* EN76 with other Cop proteins. CopC and CopD representative sequences from *Nitrosopumilus* *maritimus* (WP_012216116.1 and WP_012216034.1), *N. adriaticus* (WP_048114674.1 and WP_048114830.1), *N. piranensis* (WP_148702424.1 and WP_148702602.1), *Methylosinus trichosporium* OB3b (WP_024749324.1, WP_003610846.1, WP_003609421.1, WP_024749449.1 and WP_003609143.1), *Escherichia coli* (WP_000879316.1 and WP_000168747.1), and *Pseudomonas* *fluorescens* SBW25 (WP_015884740.1, WP_015884741.1 and WP_012722635.1) were included. The numbers above and alongside each sequence fragment indicate the residue position. The green colour in the alignments indicates the consensus positions of the copper-binding site


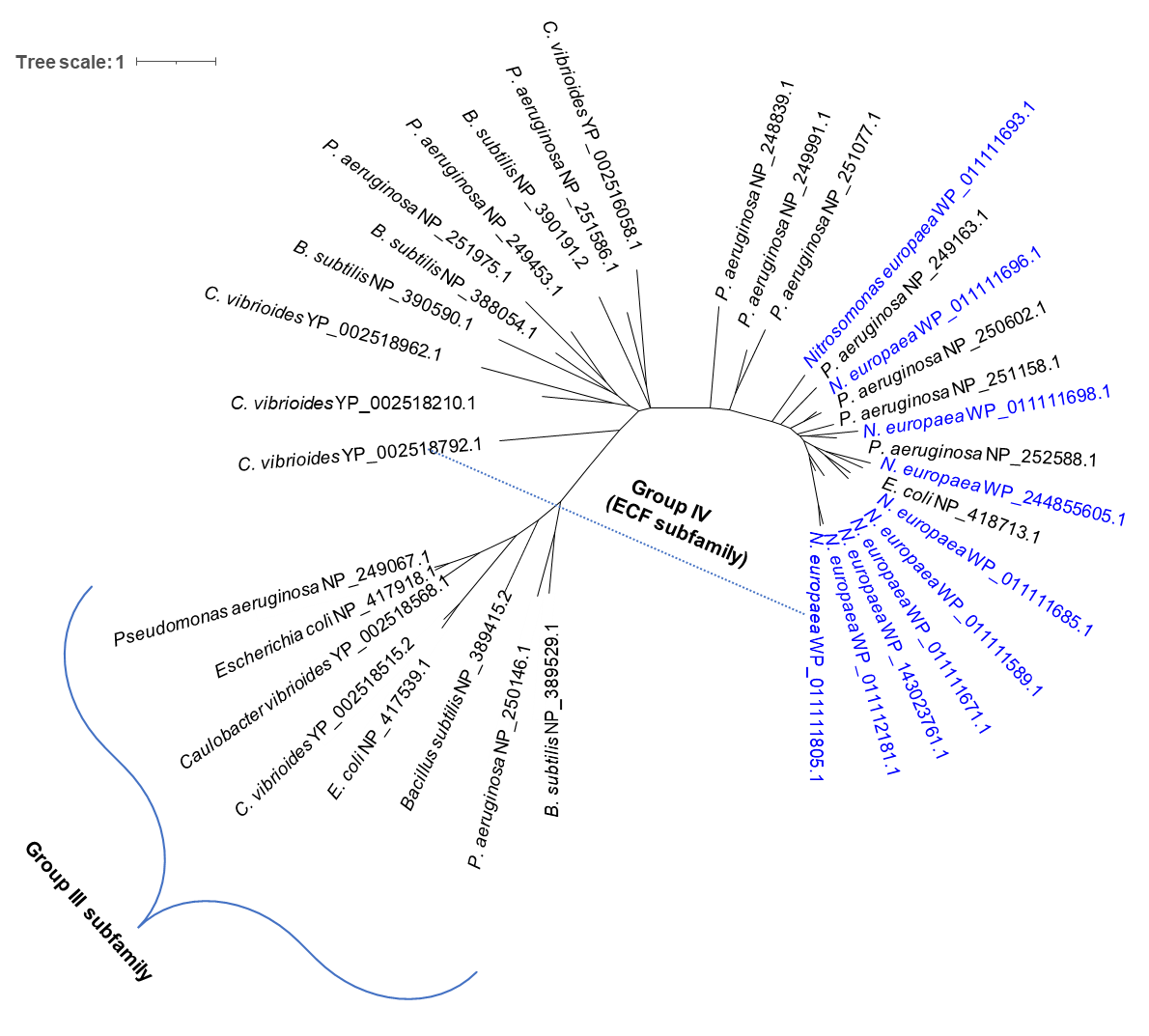


**Figure S8.** Maximum-likelihood phylogenetic tree of the nine upregulated sigma-70 protein sequences from the genome of *N. europaea* ATCC 19718 floating filter-grown cells. Representative amino acid sequences of sigma-70 protein in the genome of some other bacterial strains were retrieved from the National Center for Biotechnology Information databases. The unrooted tree was constructed with IQ-TREE (IQ-TREE options: -B 1000 -m MFP) using aligned sigma-70 protein sequences (details in *Materials and Methods*). The scale bar represents 1 change per amino acid position. The sequence ID (NCBI accession number) and the origin of each sequence are given for all strains. All the sigma-70 protein sequences from *N. europaea* ATCC 19718 are phylogenetically related to the group IV (sigma ECF) subfamily.

**Supplementary Table S1.** Effect of inoculum sizes and growth conditions on specific growth rate (*µ*_max_) of AOM strains.

| AOM | Inoculum sizes | Growth conditions | Specific growth rate (*µ*_max_) |
| --- | --- | --- | --- |
| *N. viennensis* EN76 | ~10^5^ cells | Floating filters | 0.00±0.00^c^ |
|  | ~10^6^ cells | Floating filters | 0.00±0.00^c^ |
|  | ~10^7^ cells | Floating filters | 0.23±0.01^b^ |
|  | ~10^5^ cells | Liquid media | 0.65±0.03^a^ |
|  | ~10^6^ cells | Liquid media | 0.68±0.02^a^ |
|  | ~10^7^ cells | Liquid media | 0.68±0.01^a^ |
|  |  |  |  |
| ‟*N. chungbukensis*” MY2 | ~10^6^ cells | Floating filters | 0.00±0.00^b^ |
|  | ~10^7^cells | Floating filters | 0.00±0.00^b^ |
|  | ~10^8^ cells | Floating filters | 0.00±0.00^b^ |
|  | ~10^6^ cells | Liquid media | 0.34±0.03^a^ |
|  | ~10^7^ cells | Liquid media | 0.35±0.03^a^ |
|  | ~10^8^ cells | Liquid media | 0.35±0.02^a^ |
|  |  |  |  |
| *N. europaea* ATCC 19718 | ~10^4^ cells | Floating filters | 0.71±0.01^a^ |
|  | ~10^5^ cells | Floating filters | 0.75±0.00^a^ |
|  | ~10^6^ cells | Floating filters | 0.71±0.02^a^ |
|  | ~10^4^ cells | Liquid media | 0.75±0.03^a^ |
|  | ~10^5^ cells | Liquid media | 0.75±0.02^a^ |
|  | ~10^6^ cells | Liquid media | 0.76±0.02^a^ |

The *µ*_max_ was calculated based on ammonia oxidation activity during exponential growth. Data are presented as mean ± SD (*n*=3). Significant differences between inoculum sizes and growth conditions in each strain are indicated by superscripted lowercase letters (Two-way ANOVA, Tukey’s test, *p* *<* 0.05).

**Supplementary Table S2.** Effect of CaCO_3_ particles on specific growth rate (*µ*_max_) of *N. viennensis* EN76 and ‟*N. chungbukensis*” MY2 on floating filters.

| AOM | Growth conditions | Specific growth rate (*µ*_max_) |
| --- | --- | --- |
| *N. viennensis* EN76 | Filter without CaCO_3_ | 0.00±0.00^d^ |
|  | Filter with CaCO_3_ | 0.44±0.01^c^ |
|  | Liquid media without CaCO_3_ | 0.66±0.02^a^ |
|  | Inverted filter without CaCO_3_ | 0.59±0.00^b^ |
|  |  |  |
| ‟*N. chungbukensis*” MY2 | Filter without CaCO_3_ | 0.00±0.00^b^ |
|  | Filter with CaCO_3_ | 0.00±0.00^b^ |
|  | Liquid media without CaCO_3_ | 0.36±0.00^a^ |
|  | Inverted filter without CaCO_3_ | 0.00±0.00^b^ |

The *µ*_max_ was calculated based on ammonia oxidation activity during exponential growth. Data are presented as mean ± SD (*n*=3). Significant differences between growth conditions in each strain are indicated by superscripted lowercase letters (Ono-way ANOVA, Tukey’s test, *p* *<* 0.05).

**Supplementary Table S10.** Effect of H_2_O_2_ scavengers on specific growth rate (*µ*_max_) of varying inoculum sizes of *N. viennensis* EN76 on floating filters.

| Inoculum sizes | Growth conditions | Specific growth rate (*µ*_max_) |
| --- | --- | --- |
| ~10^6^ cells | 0 mM pyruvate | 0.00±0.00^d^ |
|  | 0.1 mM pyruvate | 0.00±0.00^d^ |
|  | 1 mM pyruvate | 0.33±0.02^b^ |
|  | Catalase (10 U mL^-1^) | 0.35±0.05^b^ |
|  | 0.1 mM pyruvate (Liquid media) | 0.68±0.00^a^ |
|  |  |  |
| ~10^7^ cells | 0 mM pyruvate | 0.22±0.00^c^ |
|  | 0.1 mM pyruvate | 0.23±0.02^c^ |
|  | 1 mM pyruvate | 0.36±0.02^b^ |
|  | Catalase (10 U mL^-1^) | 0.34±0.05^b^ |
|  | 0.1 mM pyruvate (Liquid media) | 0.68±0.01^a^ |

The *µ*_max_ was calculated based on ammonia oxidation activity during exponential growth. Data are presented as mean ± SD (*n*=3). Significant differences between inoculum sizes and growth conditions are indicated by superscripted lowercase letters (Two-way ANOVA, Tukey’s test, *p* *<* 0.05).

**Supplementary Tables in Excel file**

**Supplementary Table S3:** List of all the genes expressed in *N. viennensis* EN76 grown on floating filters and liquid media.

**Supplementary Table S4:** Genes considered significantly upregulated or downregulated in *N. viennensis* EN76 grown on floating filters and liquid media. The threshold values of differentially expressed genes were set as log_2_FC > 1 and FDR < 0.05.

**Supplementary Table S5:** Differentially expressed genes involved in cell surface modification, EPS production, motility, and adhesion in *N. viennensis* EN76 grown on floating filters and liquid media. The threshold values of differentially expressed genes were set as log_2_FC > 1 and FDR < 0.05.

**Supplementary Table S6:** Differentially expressed genes involved in ammonia oxidation in *N. viennensis* EN76 grown on floating filters and liquid media. The threshold values of differentially expressed genes were set as log_2_FC > 1 and FDR < 0.05.

**Supplementary Table S7:** Differentially expressed genes involved in the electron transport chain in *N. viennensis* EN76 grown on floating filters and liquid media. The threshold values of differentially expressed genes were set as log_2_FC > 1 and FDR < 0.05.

**Supplementary Table S8:** Differentially expressed genes involved in cell division in *N. viennensis* EN76 grown on floating filters and liquid media. The threshold values of differentially expressed genes were set as log_2_FC > 1 and FDR < 0.05.

**Supplementary Table S9:** Differentially expressed genes involved in oxidative stress and inorganic nutrient homeostasis in *N. viennensis* EN76 grown on floating filters and liquid media. The threshold values of differentially expressed genes were set as log_2_FC > 1 and FDR < 0.05.

**Supplementary Table S11:** List of all the genes expressed in *N. europaea* ATCC 19718 grown on floating filters and liquid media.

**Supplementary Table S12:** Genes significantly upregulated or downregulated in *N. europaea* ATCC 19718 grown on floating filters and liquid media. The threshold values of differentially expressed genes were set as log_2_FC > 1 and FDR < 0.05.

**Supplementary Table S13**: Relative abundance of ASVs of total prokaryotes enriched on floating filters and liquid media.

**Supplementary Table S14**: Relative abundance of ASVs of nitrifiers enriched on floating filters and liquid media.

# **Supplementary References**

1. Pelve EA, Lindås AC, Martens-Habbena W, de la Torre JR, Stahl DA, Bernander R. Cdv-based cell division and cell cycle organization in the thaumarchaeon *Nitrosopumilus* *maritimus*. Mol Microbiol 2011;**82**:555–66.

2. Caspi Y, Dekker C. Dividing the archaeal way: The ancient Cdv cell-division machinery. Front Microbiol 2018; **9**:330290.

3. Heimann JD. The extracytoplasmic function (ECF) sigma factors. Adv Microb Physiol 2002;**46**:47–110.

4. Paget MS, Helmann JD. The 70 family of sigma factors. Genome Biol. 2003;**4**:203.

5. Butcher BG, Mascher T, Helmann JD. Environmental sensing and the role of extracytoplasmic function sigma factors. Bacterial physiology*:* A molecular approach. 2008, 233–61.

6. Chevalier S, Bouffartigues E, Bazire A, Tahrioui A, Duchesne R, Tortuel D, et al. Extracytoplasmic function sigma factors in Pseudomonas aeruginosa. Biochim Biophys Acta Gene Regul Mech 2019;**1862**:706–21.

7. Staroń A, Sofia HJ, Dietrich S, Ulrich LE, Liesegang H, Mascher T. The third pillar of bacterial signal transduction: Classification of the extracytoplasmic function (ECF) σ factor protein family. Mol Microbiol 2009;**74**:557–81.

8. Braun V, Hartmann MD, Hantke K. Transcription regulation of iron carrier transport genes by ECF sigma factors through signaling from the cell surface into the cytoplasm. FEMS Microbiol Rev 2022;**46**:fuac010.

9. Bashyam MD, Hasnain SE. The extracytoplasmic function sigma factors: Role in bacterial pathogenesis. Infect Genet and Evol 2004;**4**:301–8.

10. Nakamura K, Go N. Function and molecular evolution of multicopper blue proteins. Cell Mol Life Sci 2005;**62**:2050–66.
